# Recurrent patent infections with *Toxocara canis* in household dogs older than six months: a prospective study

**DOI:** 10.1186/s13071-016-1816-7

**Published:** 2016-10-04

**Authors:** Rolf Nijsse, Lapo Mughini-Gras, Jaap A. Wagenaar, Harm W. Ploeger

**Affiliations:** 1 Department of Infectious Diseases and Immunology, Faculty of Veterinary Medicine, Utrecht University, P.O. box 80.165, 3508 TD Utrecht, The Netherlands; 2 National Institute for Public Health and the Environment, Centre for Infectious Disease Control (CIb), Bilthoven, The Netherlands; 3 Central Veterinary Institute of Wageningen UR, Lelystad, The Netherlands

**Keywords:** Deworming, Dogs, Recurrent patent infections, *Toxocara canis*, Longitudinal study

## Abstract

**Background:**

To reduce environmental contamination with *Toxocara canis* eggs, the current general advice is to deworm all dogs older than six months on average four times a year. However, only a small proportion of non-juvenile household dogs actually shed *T. canis* eggs, and some dogs shed eggs more frequently than others. The identification of these frequent shedders and the associated risk factors is an important cornerstone for constructing evidence-based deworming regimens. The purpose of this study is to identify risk factors associated with recurrence of periods of shedding *Toxocara* eggs in a cohort of household dogs older than six months.

**Methods:**

We performed a prospective study (July 2011 to October 2014) on shedding *Toxocara* eggs in a cohort of 938 household dogs older than six months from all over the Netherlands. The median follow-up time was 14 months. Monthly, owners sent faecal samples of their dogs for *Toxocara* testing and completed a questionnaire. Dogs were dewormed only after diagnosis of a patent infection (PI). Survival analysis was used to assess factors influencing the time to first diagnosed PIs (FPI) and the time to recurrent PIs (RPI).

**Results:**

The overall prevalence of PIs was 4.5 %, resulting in an estimated average incidence of 0.54 PIs/dog/year. No PI was diagnosed in 67.9 % of the dogs, 17.5 % of the dogs went through only one PI and 14.6 % had > 1 PI. Prevalence of PIs always peaked during wintertime. Increased hazards for first diagnosed PIs were associated with coprophagy, geophagy, walking off-leash for ≥ 80 % of walking time, reported worms in the faeces, feeding a commercial diet and suffering from urologic or respiratory conditions. Median time to reinfection was nine months. Factors associated with increased hazards for recurrent PIs were taking corticosteroids, changing dog’s main purpose, and proxies for veterinary care-seeking behaviours.

**Conclusions:**

We concluded that targeted anthelmintic treatments in household dogs may be feasible as PIs tend to (re)occur in specific periods and in groups of dogs at high risk. Moreover, recurrent PIs appear to be influenced more by factors related to impaired immunity than environmental exposure to *Toxocara* eggs.

**Electronic supplementary material:**

The online version of this article (doi:10.1186/s13071-016-1816-7) contains supplementary material, which is available to authorized users.

## Background

*Toxocara canis* is a worldwide-distributed parasitic roundworm of canids with recognized zoonotic potential [1–4]. In patent infections, adult *T. canis* worms live in the intestine of dogs and other canids, laying eggs that pass into the faeces and contaminate the environment [5]. Within these eggs, a third-stage larva develops, after which the eggs are infective. This embryonation process usually takes several weeks [6, 7]. Like other paratenic hosts, humans can become infected by ingesting embryonated eggs or larvae in raw or undercooked meat.

In young dogs (≤ six months of age), the ingestion of infective *T. canis* eggs is most likely to lead to hepato-tracheal migration of the larvae followed by a patent infection. Conversely, the ingestion of infective eggs by older dogs (> six months of age) is less likely to lead to patent infections, as dogs develop immunity against the tracheal migration of the larvae [8, 9], resulting in so-called somatic migration [10]. This migration route leads to larvae residing somewhere in a dog’s body where they can survive for long periods, but it does not lead to a patent infection. Therefore, most dogs older than six months do not actively contribute to the environmental contamination with *T. canis* eggs. Yet, some dogs older than six months do occasionally develop patent *T. canis* infections [11]. This is likely due to insufficient levels of built-up immunity or to temporary changes in immunity, e.g. because of endocrinologic perturbations, immune disorders or stress. Also the uptake of low numbers of infective eggs [9, 11, 12] or the infection with larvae (rather than infective eggs) by consumption of raw meat and offal from infected paratenic hosts can lead to patent infections in adult dogs due to the evasion or avoidance of acquired immunity on lung level [10].

The fact that a few dogs older than six months do shed *T. canis* eggs [13–16], posing a risk for human infection, is used to justify the current ‘preventive’ three-to-four-times-a-year blind deworming advice for household dogs in this age category [17]. However, it has not yet been proved that such a treatment strategy is effective in reducing the contamination of the environment [16, 18], whilst it does lead to numerous treatments administered in absence of an actual patent infection to be treated. Therefore, monthly or three-monthly faecal examinations are also recommended as a feasible alternative to ‘preventive blind treatment’ [17]. To implement evidence-based treatment strategies for dogs, it is crucial to identify dogs that are prone to develop patent *T. canis* infections [13, 19–21]. In young dogs or in dogs infected with larvae instead of eggs, a defined prepatent period can be used for preventive treatment. For most other dogs, however, a suitable interval is less obvious because the acquired immunity will prevent the development of patent infections or prolong the prepatent period to variable extents following ingestion of infective eggs. Cross-sectional studies in North-European countries show that, at any given point in time, about 5 % of household dogs shed *T. canis* eggs in their feces [14–16, 22, 23]. However, such studies usually fail to show to what extent and at what interval dogs older than six months of age experience recurrent *T. canis* infections. Adult dogs that are frequent egg shedders are more suited targets for regular treatments. To address the occurrence of recurrent *T. canis* infections in non-juvenile dogs, we performed a longitudinal study comprising a large cohort of household dogs older than six months in the Netherlands. The aim of this study was to determine the frequency of, and factors associated with, recurrent patent *T. canis* infections in these dogs.

## Methods

### Study design and dog population

Each month for a maximum period of 40 months (July 2011 to October 2014), dog owners in the Netherlands were asked to submit a faecal sample of their dog(s) to be examined for the presence of helminth eggs (see below) at the faculty of Veterinary Medicine of Utrecht University. Along with each submitted sample, owners were asked to complete a web-based questionnaire to collect relevant epidemiological information (see below). Dog owners were enrolled *via* advertising the opportunity of enrolment in the study across pet shops, veterinary clinics, pet-themed websites and dog breed societies in the Netherlands. Additionally, flyers were handed out at some dog walking areas. Recruitment of dogs from already participating owners was allowed during the entire study period. To be enrolled in the study, dogs had to be at least six months of age and, for logistic reasons, each owner was allowed to enrol a maximum of four dogs. Laboratory results were sent monthly by e-mail to the participating dog owners. Once enrolled in the study, dogs were not allowed to be dewormed unless a positive laboratory result was obtained, the dogs were traveling to *Dirofilaria immitis*-endemic areas, or they were lactating and performing litter care. In case of a positive laboratory result, the owners were asked to prevent their dog from eating anything from the ground for at least 3 days and send in a new sample. This step was included to rule out positive samples due to coprophagy as much as possible [24, 25]. If this confirmation sample tested positive also, it was considered a patent infection. After a positive confirmation sample, a short-acting anthelminthic product (containing febantel, pyrantel and praziquantel) was provided. If a parasitic infection (e.g. *Cystoisospora* spp.) was diagnosed that could not, either legally or due to suboptimal efficacy, be cured with this anthelminthic, owners were advised to confer with their veterinarian.

Owners participated in this study knowing that the acquired data would be used for a scientific publication.

### Collection of epidemiological data

Epidemiological data were collected *via* a self-administered questionnaire that could be answered online. We differentiated between the starting questionnaire (completed at submission of the first faecal sample) and the follow-up questionnaires, which were completed at submission of each subsequent sample. The starting questionnaire contained questions about the dog's age, sex, breed, function, reproductive status, living conditions, diet, time roaming freely, predatory and coprophagic behavior, health status, medication use, and deworming history. The follow-up questionnaires were meant to monitor any change in living conditions, lifestyle (e.g. diet, function, etc.) or health of the dogs relative to the preceding questionnaire. Owners were specifically asked to report whether and when their dogs had been dewormed for reasons other than those provided above. A copy of the questionnaires is available as supplementary data (Additional file 1). Information on socio-economic status (SES, a normalized score ranging from - 4 to + 4 based on income, employment and educational level per postcode area) and urbanization degree (> 2,000, 1,500–2,000, 1,000–1,500, 500–1,000, and < 500 addresses/km2) was obtained at the postal code level from Statistics Netherlands (http://www.cbs.nl/en-GB/menu/home/default.htm).

### Coproscopical examination

Samples were submitted individually from each dog using a collection box at the faculty of Veterinary Medicine of Utrecht University (for people living or working close by) or submitted to the laboratory by regular mail, using study-provided materials and instructions. Each sample was identified by a unique code, which was linked to the questionnaire. The centrifugal sedimentation and flotation technique used for coproscopical analysis has been reported previously [16, 25, 26]. For each sample, at least three grams of faeces was used and a sugar solution (s.g. 1.27–1.30 g/cm^3^) was used as flotation medium. This method has a theoretical detection limit of detecting 1.6 eggs per gram. Slides were microscopically examined at 40×, 100× and 400× magnification. *T. canis* eggs were measured and morphologically identified, using the AAVP reference guide for diagnosing parasitism in animals [27]. For logistic reasons, two samples (three grams each) were pooled in the laboratory for first testing, with a theoretical detection limit of 3.2 eggs per gram for each individual dog in the pooled sample. If this pooled sample tested positive for dog-typical parasites, the samples were re-tested separately to determine which sample contained the eggs.

### Data analysis

Survival analysis was used to assess factors influencing the time to the “first” diagnosed event of *Toxocara* egg shedding (first patent infection = FPI) and the time to recurrence of a patent infection (recurrent patent infection = RPI) in our dog population. This was done using Cox proportional hazards models, which assessed the risk of patent *Toxocara* (re)infection longitudinally as a function of the factors measured at each sampling event. For the time to FPI, dogs entered the cohort at the submission of the first sample and were censored at their first diagnosed infection. Observation time for the time to FPI was then calculated as the time from the submission of the first sample (i.e. enrolment in the study) to that of the FPI or the end of the follow-up period (i.e. end of study or dropout from study). For the time to RPI, entry into the cohort began with the FPI and dogs were not censored after each subsequent reinfection. A conditional risk set model [28], in which the analysis is stratified by event (i.e. infection) order, was used for the analysis of the time to RPI. The assumption is that the conditional risk at time *t* for event *k* derives from all subjects under observation at time *t* that have had event *k* - 1. The method is widely used for analysis of recurrent events in the biomedical literature [29]. Observation time for the time to RPI was then defined as the gap time between subsequent infections (i.e. time to each event is measured from the previous event), or from the FPI to the end of the follow-up period (end of study or dropout from study) if the dogs did not have a RPI. Associations were expressed as hazard ratios (HRs) with 95 % confidence interval (95 % CI).

Preliminary analyses included log-rank tests for equality of survivor functions and Kaplan-Meier curves to assess graphically the assumption of proportionality for Cox proportional hazards for each independent variable. Variables satisfying these conditions were selected for inclusion in a multivariable Cox proportional hazard regression model. A backward stepwise selection procedure was then applied, with variables showing a *P* ≤ 0.05 for the association with the outcome variable being retained in the model. The effect of removing variables on the associations of the other covariates was also monitored. A change of ≥ 10 % in the coefficients was considered as a sign of confounding and the variable in question was retained in the model regardless of significance. The variables dog’s age (6–12 months, 1–7 years, > 7 years), sex, season (winter, December-February; autumn, September-November; spring, March-May; summer, June-August), time since last deworming (continuous variable expressed in months) and reported coprophagic behaviour were always controlled for in the models. The tested variables are intrinsic to the questions in the questionnaire (Additional file 1). The SES was included as test variable, obtained at postcode level. Biologically plausible interactions between covariates were also assessed and the final model was expanded to include significant interaction terms, if any. Besides the repeated measurements made on the same dogs over time (multiple-record-per-subject analysis), we accounted for clustering (or non-independence) of dogs living in the same household (i.e. having the same owner) by incorporating cluster-robust variance estimators. Statistical analysis was performed using STATA 13 (StataCorp LP, College Station, USA).

## Results

### Descriptive statistics

**Fig. 1:**
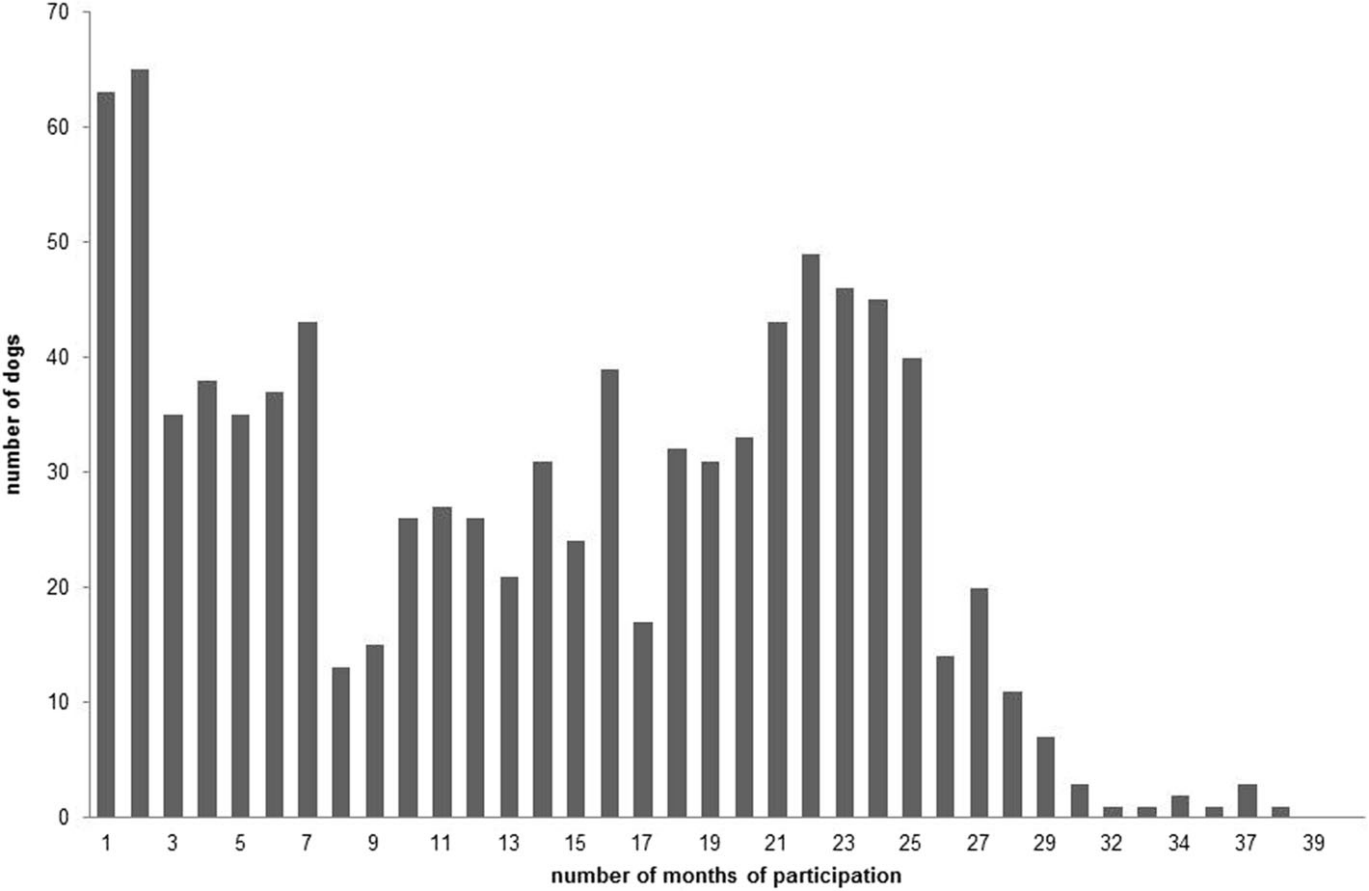
Distribution of dogs over duration of follow up. The distribution of duration of participation in months with the corresponding number of dogs

In total, 938 dogs belonging to 570 owners were enrolled in the study. The cohort was followed for a total of 12,968 dog-months. Figure 1 shows the distribution of dogs over the number of months of follow-up. The median follow-up time per dog was 14 months (interquartile range [IQR] 5–22 months). The median age of the dogs at enrolment was 4 years (IQR 2–7 years). The study population consisted of 406 (43.3 %) males and 532 (56.7 %) females (male/female ratio = 0.76).

Of 12,968 stool samples tested, 585 were positive for *Toxocara* eggs, resulting in an overall proportion of 4.5 % (95 % CI 4.0–5.1 %) positive samples. Table 1 shows the number of dogs and corresponding number of samples stratified by their number of positive test months diagnosed during the study period. In total, 301 (32.1 %) dogs had at least one *Toxocara* infection, whereas the remaining 637 dogs (67.9 %) never tested positive. The incidence rate was estimated at 0.54 patent *Toxocara* infections (95 % CI 0.48–0.61) on average per dog/year. Anthelmintic treatment was given in 84 occasions for reasons unrelated to the study (e.g. foreign travel), in these cases dogs were allowed to continue their enrollment in the project.

**Table 1:** Dogs and samples stratified by number of *Toxocara* eggs positive test months

Number of patent infections	Number of dogs (%)	Samples			
		*n*	*Toxocara* negative	*Toxocara* positive	Mean number of samples per dog
0	637 (67.9)	7,706	7,706	0	12
1	164 (17.5)	2,761	2,597	164	17
2	66 (7.0)	1,188	1,056	132	18
3	33 (3.5)	566	467	99	17
4	18 (1.9)	347	275	72	19
5	9 (1.0)	174	129	45	19
6	8 (0.9)	164	116	48	21
8	2 (0.2)	38	22	16	19
9	1 (0.1)	24	15	9	24
Total	938 (100)	12,968	12,383	585	

**Fig. 2:**
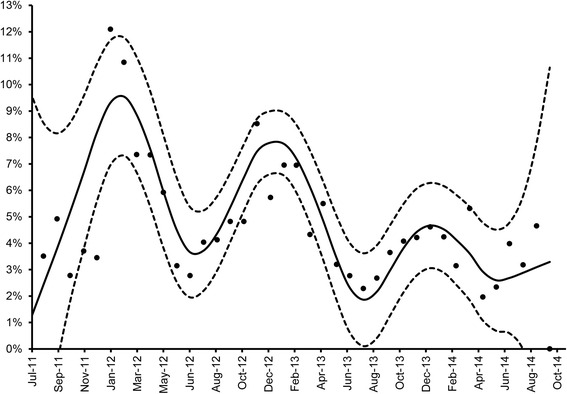
Monthly *T. canis* incidence (dots) over the study period (from July 2011 to October 2014). An optimized cubic smoothing P-spline function (solid line) and corresponding 95 % confidence interval (dotted lines) is fitted to the observed data

The monthly *Toxocara* incidence rate showed a clear seasonal pattern (Fig. 2), peaking during the winter and decreasing during the summer. Figure 2 also shows a decreasing trend in the incidence over the years.

### Survival analysis

#### Time to “first” infection

Survival analysis for the time to FPI was based on 836 dogs with observations not ending on entry or beginning on FPI. These dogs accounted for a total of 8,783 dog-months at risk under observation during which 259 FPI occurred, resulting in an incidence rate of 2.9 FPIs per 100 dog-months (95 % CI 2.6–3.3). Median time to FPI was 5 months (IQR 2–10).

The final multivariable Cox proportional hazards model for *Toxocara* FPI (Table 2) showed that the risk of observing a FPI was higher for dogs displaying coprophagic behavior or eating sand/soil, dogs ranging off-leash > 80 % of their walking time as compared to dogs ranging freely ≤ 20 % of their walking time, dogs whose owners had noticed worms in their dogs’ faeces, dogs fed with a commercial diet and dogs with urologic or respiratory conditions. The risk of having a FPI was also significantly higher in winter and autumn as compared to summer, and it increased with increasing time since last deworming. Conversely, older age groups, having neurologic conditions, and being fed with a diet containing frozen raw meat had a lower risk.

**Table 2:** Results of the final multivariable Cox proportional hazards regression model for “first” *T. canis* infection

	No. of dogs	No. of dog-months at risk	No. of observed FPIs	HR	95 % CI		*P*-value
Age group							
0–12 months	202	636	42	Ref.			
1–7 years	575	5,678	157	0.47	0.32	0.67	< 0.0001
> 7 years	250	2,469	60	0.40	0.26	0.61	< 0.0001
Sex							
Male	362	3,702	113	Ref.			
Female	481	5,081	146	0.93	0.72	1.20	0.580
Coprophagy							
No	475	4,877	115	Ref.			
Yes	400	3,906	144	1.36	1.05	1.77	0.021
Sampling season							
Summer	655	2,332	38	Ref.			
Winter	741	2,434	89	1.73	1.14	2.61	0.009
Autumn	564	1,978	76	1.62	1.05	2.50	0.030
Spring	603	2,039	56	1.28	0.81	2.01	0.287
Eating soil/sand							
No	740	7,818	213	Ref.			
Yes	106	965	46	1.62	1.12	2.35	0.011
Following a commercial diet							
No	320	3,081	68	Ref.			
Yes	567	5,702	191	1.47	1.08	2.00	0.014
Following a diet containing frozen raw meat							
No	381	3,377	131	Ref.			
Yes	494	5,406	128	0.68	0.52	0.89	0.005
Having respiratory conditions							
No	822	8,456	244	Ref.			
Yes	46	327	15	1.84	1.08	3.13	0.026
Having neurologic conditions							
No	819	8,413	257	Ref.			
Yes	40	370	2	0.21	0.06	0.82	0.024
Having urologic conditions							
No	816	8,467	245	Ref.			
Yes	41	316	14	1.79	1.12	2.86	0.015
Excreting worms in faeces							
No	828	8,688	252	Ref.			
Yes	10	95	6	2.26	1.16	4.42	0.017
Off-leash walking time (%)							
≤ 20	131	1,325	29	Ref.			
20–50	260	2,461	54	1.06	0.64	1.75	0.829
50–80	141	1,337	40	1.30	0.76	2.23	0.341
> 80	375	3,660	136	1.79	1.13	2.83	0.013
Time since last deworming (months)^a^	–	–	–	1.002	1.000	1.003	0.024

^a^Continuously time-varying variable let interact with the underlying time variable

*Abbreviations*: *HR* hazard ratio; 95 % CI, 95 % confidence interval; *FPI* “first” patent infection; *Ref*. reference category

#### Time to reinfection

Time to RPI analysis was based on 281 dogs in which a FPI was diagnosed and from which subsequent samples were submitted. The corresponding incidence rate of 284 reinfections over 3,247 dog-months at risk under observation was 8.7 RPIs per 100 dog-months (95 % CI 7.7–9.7). Median time to RPI was 9 months (IQR 3–16).

The multivariable Cox proportional hazards regression model for *Toxocara* reinfection (Table 3) showed that the risk of reinfection was significantly higher for dogs receiving corticosteroid treatment, for dogs whose main purpose/use was changed, and for dogs whose owners reported that they would usually buy anthelmintic drugs at veterinary clinics. The risk of reinfection was also significantly higher in winter as compared to the summer, and it increased with increasing time since last deworming. Conversely, the risk of RPI was significantly lower for dogs whose owners reported to sometimes or always collect and dispose of their dogs’ faeces as compared to those who reported to never do that, as well as for dogs with neurologic conditions, and was borderline significant for dogs with orthopedic conditions.

**Table 3:** Results of the final multivariable Cox proportional hazards regression model for *T. canis* reinfection

	No. of dogs	No. of dog-months at risk	No. of observed RPIs	HR	95 % CI		*P*-value
Age group							
6–12 months	49	110	30	Ref.			
1–7 years	202	2,211	170	0.85	0.49	1.46	0.552
> 7 years	96	926	84	0.87	0.47	1.62	0.663
Sex							
Male	121	1,400	125	Ref.			
Female	162	1,847	159	1.27	0.91	1.77	0.160
Coprophagy							
No	118	1,234	84	Ref.			
Yes	170	2,013	200	1.09	0.74	1.59	0.674
Sampling season							
Summer	237	777	57	Ref.			
Winter	220	612	95	1.68	1.15	2.46	0.008
Autumn	239	857	50	1.30	0.82	2.08	0.266
Spring	244	1,001	82	1.43	0.96	2.14	0.079
Taking corticosteroids							
No	265	3,036	263	Ref.			
Yes	25	211	21	2.38	1.09	5.19	0.029
Frequency of dog’s faeces removal/disposal							
Never	32	351	48	Ref.			
Sometimes	160	1,903	164	0.54	0.33	0.86	0.01
Always	89	993	72	0.54	0.32	0.91	0.02
Change in dog’s main purpose/use							
No	280	3,238	281	Ref.			
Yes	8	9	3	10.84	1.14	103.21	0.038
Having neurologic conditions							
No	279	3,188	283	Ref.			
Yes	9	59	1	0.11	0.02	0.74	0.023
Having orthopaedic conditions							
No	262	2,891	260	Ref.			
Yes	45	356	24	0.55	0.28	1.06	0.074
Owner usually buys anthelmintic drugs at veterinary clinics							
No	134	1,464	107	Ref.			
Yes	147	1,783	177	1.52	1.10	2.11	0.011
Time since last deworming (months)^a^	–	–	–	1.003	1.001	1.005	< 0.0001

^a^Continuously time-varying variable let interact with the underlying time variable

*Abbreviations*: *HR* hazard ratio; 95 % CI, 95 % confidence interval; *RPI* recurrent patent infection; *Ref.* reference category

## Discussion

Longitudinal studies are better suited than cross-sectional studies to investigate events that can recur throughout an individual’s life. Taking the limitations of coproscopical examination to diagnose patent *Toxocara* infections into account [30], our study reports an estimated prevalence of 4.5 % dogs that shed *Toxocara* eggs, which is comparable with reported, mostly cross-sectional, prevalences from current literature [14–16, 22, 23]. Monthly incidence ranged from 2 to 12 %, peaking consistently during wintertime in all three years of follow-up. This finding was unexpected, as one would hypothesize that in a country like the Netherlands where seasons are well defined, dogs are walked outdoor for longer periods (and perhaps more often unleashed) during the summer as compared to winter because of the generally more favorable/pleasant weather conditions, and this would impose a higher risk for infection. Yet, similar seasonal patterns were noted by others [15, 31, 32]. Although no exhaustive explanation can be provided, it is evident that the winter peaks were consistently present in all three years of follow-up. The possibility that the observed seasonal pattern reflects more frequent deworming in summertime could be ruled out because the surveyed dog population was not routinely dewormed, as this was a condition for participation in the study. Wolves (*Canis lupus*), the far ancestors of dogs, are mono-estrus species that breed in mid to late winter, and the associated endocrinological changes might reactivate dormant *T. canis* larvae during that period as is known in dogs. It is likely that the change in day length is the stimulus of this breeding cycle. We speculate that in the co-evolution of the parasite and its definitive host, this phenomenon might have persisted even though household dogs do not necessarily show a well-defined seasonal breeding pattern any longer [33]. However, kenneled cyclic beagles were not at higher risk for developing a patent *T. canis* infection, which makes this hypothesis less likely [34]. An additional explanation might be that shorter walks and staying longer at home during wintertime may act as stressor, contributing to reactivation of dormant larvae. Reactivation of dormant somatic larvae is likely to be responsible instead of an increased risk of being re-infected by ingestion of infective eggs. Another possible stressor could be related to the intensive use of fireworks during the festive period in the Netherlands in the last months of the year. A recent study reported increased cortisol levels, a common indicator of stress, in dogs during winter [35]. Whatever the reasons might be, it is apparent that seasonality in *T. canis* egg shedding exists and needs to be considered in future studies, especially when these are performed cross-sectionally at one moment in time. Moreover, understanding the origin of this seasonal pattern is relevant for control, as any (blind) deworming would be more likely to be necessary during the coldest rather than the warmest months. Besides seasonality, monthly *Toxocara* incidence also tended to decline over time even though no blind deworming was applied. This may be explained by the aging of the cohort and by the loss of follow-up of some frequent shedders.

Most (67.9 %) participating dogs were never diagnosed with a patent *T. canis* infection during the follow-up period, 17.5 % dogs experienced only one infection, and 14.6 % dogs experienced two or more infections, with a maximum of nine patent infections diagnosed in the same dog during a follow-up period of 24 months. Based on the observed frequency of infection, the average annual incidence rate was estimated at 0.54 patent infections/dog/year, which can be translated into one infection occurring approximately every two years among household dogs that are not (blindly) treated on a regular basis. Consequently, it could be said that the currently propagated four-times-a-year anthelmintic treatment advice lacks evidence for dogs older than six months. Our data suggest that targeted treatments may be preferable over blind treatments. A two-step approach was applied in the longitudinal analysis. First, a survival analysis for identifying factors influencing the time to FPI was performed. Secondly, survival analysis was performed for identifying factors influencing the time to RPI. The time to FPI was measured from the moment of enrollment till the first diagnosed patent infection, without knowing when these dogs had actually experienced the previous patent infection before participating in the study. In general, similar risk factors for FPI were found in the present study compared to previous (cross-sectional) studies [13, 19–21], as well as to a previous cross-sectional study based on the same dog population, where only the first submitted sample of a dog was included, as in the present study [16]. These were young age, coprophagy, and proportion of walking time walking off-leash. An unexpected risk factor was the feeding of a commercial diet, while feeding frozen raw meat appeared to be protective. Though most of the *T. canis* larvae present in the meat will be killed by freezing it, this is not always the case [36–38]. The assumption that raw meat may be a risk factor for *T. canis* infection is only valid if the meat in question contains dormant larvae. The origin of the consumed meat is therefore important to take into account, as meat from farms with a high level of biosecurity is highly unlikely to contain dormant larvae. Yet, previous research indicated that owners feeding raw meat to their dogs do not often know the origin of that meat (unpublished data). Explaining that commercial (bagged/canned) diets are a risk factor for patent *T. canis* infections is difficult. It is important to realize that this association might just be a spurious one due to a hitherto unknown confounder that was not accounted for in the analysis. We speculate that dogs receiving a commercial diet, which is usually easier for dogs to eat, might be more prone to the need of chewing/gnawing items, perhaps from the ground outside increasing the risk of infection. However, estimates were adjusted for “eating soil/sand” (included as covariate in the model) and were not influenced by the factor “eating items from the ground” (not significant). Interestingly, in the analysis of recurrent infections, factors related to diet were not associated with testing positive on *Toxocara* eggs. Eating soil/sand turned out to be a risk factor for FPI. This suggests that infective eggs are ingested, as coprophagy was controlled for in the analysis, or that eggs passively pass the gastro-intestinal tract after eating soil/sand. Normally, this would not lead to patent infection in adult dogs. However, it has been reported that infection with low numbers of eggs may sometimes lead to patent infection, as low numbers of larvae may pass undetected by the host’s immune system during their hepatic-tracheal migration [12]. The observed effects of some health conditions on the risk of *Toxocara* egg shedding may be a reflection of the stress induced by the conditions themselves and/or by the decreased immune-competence that these conditions may entail. In contrast, having neurologic or orthopedic conditions stood out as a protective factor. Dogs with these conditions tend to be less active outside, thereby reducing the risk of acquiring a *T. canis* infection from the environment, which may oppose an effect of stress induced by these conditions.

The analysis of RPIs showed an incidence of 8.7 reinfections/100 dog-months, more than three times that for FPIs. This suggests that recurrent shedding of *Toxocara* eggs occurs more often in some dogs that for some reason are particularly prone to experience multiple patent *T. canis* infections. Such dogs may be called “wormy” dogs and, hence, should be a specific target for treatments. This group of “wormy” dogs is responsible for the majority (421 of 585 or 72 %) of positive faeces samples (see Table 1).

Determining factors associated with (recurrent) infections in these dogs would therefore provide useful targets for control. The factors associated with RPIs found here showed some overlap with those for the FPIs. However, there were also some interesting differences, which mainly concerned factors mirroring the immunological status of the dog. For instance, the administration of corticosteroids, known for their immunosuppressive action, resulted in a HR of 2.38 for experiencing a RPI. Sudden changes in the routine of the dog (i.e. main purpose or use of the dog), which may well lead to a temporarily suboptimal immune status due to stress, resulted in a hazard ratio of 10.84. The latter becomes even more plausible when having a closer look at the data (results not shown), as the owners whose dogs had their purpose changed mostly reported that their dogs had become hunting dogs. It is known that hunting activities can be quite stressful for dogs [39]. Previously identified risk factors for patent *Toxocara* infection may also be explained, to some extent, by (temporary) perturbations of the immune status, such as being kenneled [16]. This implies that dogs under periods of stress are at risk of becoming shedders of *Toxocara* eggs and should be targeted by anthelmintic treatment.

Cleaning up dogs’ faeces by owners appeared to be protective for RPIs. Although such behavior in a dog owner is unlikely to be directly related to the risk of infection in the respective dog, it may mirror a general habit of disposing of dogs’ faeces in the area where the owner lives, and therefore to a societal pressure to clean up dogs’ faeces, possibly resulting in a generally less contaminated environment with *T. canis* eggs shed by dogs. Coprophagy was identified as a risk factor for FPI, as well as in a previous cross-sectional study [16], but it was no longer significant for RPIs. This suggests that *T. canis* eggs in the faeces of dogs showing recurrent infections are more likely to be eggs from an actual infection rather than eggs simply passing the gastrointestinal tract after ingestion of unembryonated eggs with the faeces of real *T. canis* shedders. In contrast, dogs incidentally shedding *Toxocara* eggs may often do so because of coprophagy. Coprophagy is a possible factor that can influence the outcome of coproscopical examinations and when performing such methods it should be considered when an animal tests positive [24, 25]. Finally, buying anthelmintics at the veterinary clinic was a risk factor for RPIs. This is hard to explain by simply looking at the biology of the parasite or the host. However, because anthelmintics in the Netherlands can also be purchased (sometimes for cheaper prices) at pet stores, internet, supermarkets, and department stores, owners buying anthelmintics at veterinary clinics do so probably because they happen to frequently visit the clinic for the health problems of their dogs, so this factor may simply mirror frequent veterinary care-seeking behaviors because of impaired health in the dogs.

## Conclusions

Following a large cohort of dogs, all older than six months, up to three years without performing routine deworming in absence of a confirmed diagnosis revealed that approximately 68 % of dogs never tested positive for *Toxocara* eggs*.* The overall incidence rate was 0.54 patent *Toxocara* infections/dog/year, meaning that a non-routinely treated dog is likely to shed *Toxocara* eggs once every two years, on average. However, the incidence rate of RPIs was much higher than that of FPIs, suggesting that there is a group of dogs particularly prone to recurrence of patent *Toxocara* infections. Dogs with RPI were responsible for the majority of positive faeces samples.

The identified risk factors for FPIs and RPIs indicate that there are two important aspects to consider when assessing the risk for a dog to acquire a *Toxocara* infection, the exposure to sources of infection and the failure of immunity. Indeed, both the likelihood of ingesting infective eggs/larvae and the possible evasion of immunity, perhaps by already present somatic larvae, should be taken into account when controlling *T. canis* infections in household dogs. Based on our study, this can be indicated by factors related to immune suppression, e.g. administration of immunosuppressive drugs or stress caused by underlying diseases or changes in routine, as well as factors related to higher chances of ingesting *T. canis* eggs from the environment, e.g. eating soil/sand or enjoying a high amount of off-leash walking time. Future modelling papers may benefit from studies that report on risk factors, especially when studied in a longitudinal set-up, so different scenarios can be tested by varying the exposure to different factors over time. Together with the observed peaks of *Toxocara* incidence during the winter months, our results suggest that blind deworming may be refined to become a more targeted deworming strategy based on the identified risk factors.

## Additional file

Additional file 1: Questionnaire. (PDF 483 kb)

## Abbreviations

PI: Patent infection
FPI: First diagnosed patent infection
RPI: Recurrent patent infection
