# Supplementary material for: Recurrent patent infections with Toxocara canis in household dogs older than six months: a prospective study
Source: Parasit Vectors. 2016 Oct 4;9:531. doi: 10.1186/s13071-016-1816-7 (PMC5051026; doi:10.1186/s13071-016-1816-7)
Supplement: Additional file 1: — Questionnaire. (PDF 483 kb) [file 13071_2016_1816_MOESM1_ESM.pdf]

Questionnaire Toxocara-study Dog – Intake

Note: all questions asked for each dog have been listed below. On the internet site there were welcome pages included, as well as that followed paths could differ depending on answers provided for specific questions. For instance, a question concerning pregnancy was only asked if an owner entered the dog was female.

Welcome to this questionnaire

Welcome to this questionnaire.
If a question is preceded by an asterisk, this means you have to answer this question to proceed to the next question.
In case of the possibility to enter multiple answers, this will be indicated.

Q: * Enter your participation ID: ………………………………

Demographic data

Please enter your personal data below.
This information is needed to send you the required envelopes and submission materials and, if necessary, anthelmintic following the finding of parasite eggs. It is also needed to contact you when required. This information will only be used for this study!
Fields marked with an asterisk are required fields.

Q: * Name: ……………………………………
Q: * Address: ……………………………………
Q: * City: ……………………………………
Q: * Postal code ……………………………………
Q: * e-mail: ……………………………………
Q: Phone: ……………………………………

Q : * How would you describe your living environment?
Explanation:
Residential area – mainly streets and houses with patches of green
Rural – little forest, predominantly farming area
Woody – predominantly forest area
Other – combinations of the above

- ❑ **residential area**

Utrecht University

- ❑ **rural**
- ❑ **woody**
- ❑ **other, namely** ……………………………….

Information about your dog.

These questions are about your dog. If chipnumber (ID chip) or breed are unknown, please enter XXX.
The name of your dog is required because some participate with up to 3 dogs.
If you have more than 1 dog, start with one. The other dogs can be entered later in this questionnaire.

Q: * Name of dog: ………………………………..

Q: Chipnumber: ………………………………..

Q: Breed: ………………………………..

Q: * Gender of dog: ❑ **male** ❑ **female**

Q: * Is your dog castrated or sterilized? ❑ **No** ❑ **Yes**

Q: * If female, is your dog pregnant (in pup)? ❑ **No** ❑ **Yes**

Q: What was the service date (conception date)? ………………………………..

Q: When was the dog in heat for the last time? ………………………………..

Q: * What is your dog's age?

*** Tickboxes ranged from 1 year to older than 13 years**

Q: Please also enter the date of birth if known: ………………………………..

Q: * For what reason do you keep your dog?

- ❑ **for companionship**
- ❑ **as yard dog**
- ❑ **hunting dog**
- ❑ **guard dog**
- ❑ **other, namely:** ………………………………..

Q: * Did your dog(s) stay in a kennel for some time during the last 2 months? ❑ **No** ❑ **Yes**

Information about other animal species in the household.

Q: * Are there any other animals than this dog in your household? ❑ **No** ❑ **Yes**

Q: Please enter which animal species? (multiple answers possible)

Tickboxes included: dog, cat, bird, rabbit, guinea-pig, reptile, other (namely …).

Behaviour of the dog

Q: * While walking the dog, is the dog sometimes loose (runs freely around)? ❑ **No** ❑ **Yes**

If yes **Q: * What percentage of the time does your dog runs loose?**

Utrecht University

Tickboxes from 10% to 100% in 10% intervals

Q: * Does your dog catch prey animals sometimes (mice, birds, etc)?

❑ **No** ❑ **Yes** ❑ **Unknown**

If yes **Q: Which animal species are captured? (multiple answers possible)**

Tickboxes included: mice, rats, rabbits, birds, frogs, other (namely ...).

Q: Does the dog eat parts of the captured prey animal?

Space to enter text for participant.

Q: * Does your dog eat stuff out of the environment (street, forest, park, etc)? ❑ **No** ❑ **Yes**

If yes **Q: What kind of stuff does your dog eat? (multiple answers possible)**

❑ **Poo/faeces**
❑ **Grass**
❑ **Sand**
❑ **Other, namely …………………………**

Q: Does your dog roll over its back on the ground outside (in park or forest)? ❑ **No** ❑ **Yes**

Food

Q: * I feed my dog (multiple answers possible)

❑ **prepackaged dogfood from a can or a bag**
❑ **fresh raw meat**
❑ **raw meat that has been frozen**
❑ **the same as what I eat as owner**
❑ **vegetarian food**
❑ **combination of the above / other, namely ……………………….**

Q: * Do you give your dog snacks in between regular meals? ❑ **No** ❑ **Yes**

If yes **Q: What do you give your dog? (multiple answers possible)**

❑ **prepackaged dog snacks**
❑ **fresh raw meat food**
❑ **raw meat food that has been frozen**
❑ **the same snacks as I take (things like nuts, cheese, cookies,etc)**
❑ **vegetarian food**
❑ **a combination / other, namely ……………………**

Health

Utrecht University

Q: * Does your dog have health issues?

Explanation:

NO: speaks for itself.

YES: means there are symptoms of disease (eg. diarrhea, itching, breathing difficulties etc.), but also medicin use (now and in the past) may indicate health issues.

- ❑ **there are NO indications of disease or suboptimal health**
- ❑ **YES, there indications of disease or suboptimal health**

If yes **Q: Which indications? ……………………………..**

Q: * Is your dog currently on medication? ❑ No ❑ Yes

If yes **Q: Which medicins are given to your dog?**

Options to enter medicin and productname for three medicins

If yes **Q: Give dose and frequency of use of medicin**

Again three textboxes to enter for the above given medicins

Q: Did your dog receive any OTHER medicins over the last 3 months? ❑ No ❑ Yes

Q: Did your dog get medication the last 3 months? ❑ No ❑ Yes

If yes **Q: What medication did your dog get the last 3 months?**

Options to enter three medications

Walking the dog

Q: * Identify location of the two most frequented areas where you walk your dog (mention streetname and city or name of park or area and city, etc).

Two textboxes for entering locations

Q: * Indicate for each location if you keep your dog mainly on a leash or let the dog roam freely some of the time

Area 1: ❑ roam freely ❑ on a leash

Area 2 ❑ roam freely ❑ on a leash

Q: * Do you dispose of the feces of your dog by picking it up from the ground?

❑ Always ❑ Sometimes ❑ Never

Q: Did you notice anything out of the ordinary of the dog's feces during the last month?

❑ No ❑ Yes

If yes **Q: What did you notice? (multiple answers possible)**

Provided options were: lighter or darker color, more fluid or firmer consistency, mucus in the feces, blood in the feces, visible worms in the feces, other (namely …..)

Deworming practices

Utrecht University

Q: * How often do you deworm your dog per year?

- ❑ **never**
- ❑ **less than once a year**
- ❑ **once a year**
- ❑ **twice a year**
- ❑ **three times a year**
- ❑ **four times a year**
- ❑ **other, namely ………………………….**

Q: When was your dog dewormed the last time? ………………………………….

Q: * Which deworming product(s) do you use?

Two text boxes available following the remark: if you use more than 1 different products, please enter the 2 products used most often

Q: * Where do you purchase the deworming product for your dog? (Multiple answers possible)

- ❑ **veterinary practice**
- ❑ **pet store**
- ❑ **garden shop (large garden centre)**
- ❑ **internet**
- ❑ **supermarket / big department store**
- ❑ **other, namely …………………………………….**

Q: * Did you dog had worm infection(s) in the past? ❑ **No** ❑ **Yes**

If yes **Q: * How long ago?**

- ❑ **one month ago**
- ❑ **half a year ago**
- ❑ **one year ago**
- ❑ **more than 1 year ago**

Q: If known, which worm infection did the dog have (eg. roundworms, tapeworms, etc) ………………………………………….

Q: * What is according to you the most important reason to deworm your dog?

- ❑ **health of my dog**
- ❑ **public health**
- ❑ **because I have to deworm**
- ❑ **other, namely ……………………………………..**

Finally

Utrecht University

Q: * Do you have 2 or more dogs participating in this study? ❑ No ❑ Yes

If yes, the participant is asked to enter the questionnaire above again for the second and possibly third dog. A maximum of three dogs per participant was allowed.

Utrecht University

Questionnaire Toxocara-study Dog – Monthly repeated

Following each monthly submitted faeces sample, participation was mailed a link to a website to enter the monthly repeated questionnaire whether there had been any changes since the last time (i.e. during the last month).

This part starts with a question whether participant moved house or not, and if so, to where including providing all new demographic data for the new living area. Subsequently, all questions up to deworming practices were repeated concerning changes. If no changes had occurred participant was guided through this repeat questionnaire quickly. The only questions not asked again concerned, chipnumber, breed and age of the dog.

All questions pertaining to deworming practices were not asked again, but were replaced by the following questions.

Q: * Did you had to deworm your dog for this study? ❑ **No** ❑ **Yes**

If yes **Q: When was your dog dewormed? ………………………………..**

Q: What product did you use for the deworming? ………………………………..

Again, the entire repeat questionnaire had to be entered for each dog separately up to 3 dogs per participant.

Utrecht University
